# Computer Analysis of the Porous Structure of Activated Carbons Derived from Various Biomass Materials by Chemical Activation

**DOI:** 10.3390/ma14154121

**Published:** 2021-07-23

**Authors:** Mirosław Kwiatkowski, Elżbieta Broniek

**Affiliations:** 1 Department of Fuel Technology, Faculty of Energy and Fuels, AGH University of Science and Technology, al. A. Mickiewicza 30, 30-059 Krakow, Poland; 2 Department of Chemistry and Technology of Fuels, Faculty of Chemistry, Wrocław University of Technology, Gdańska 7/9, 50-344 Wrocław, Poland; elzbieta.broniek@pwr.edu.pl

**Keywords:** biomass, adsorption, porous structure, chemical activation, activated carbons

## Abstract

In this study, the preparation of activated carbons from various materials of biomass origin by activation with potassium hydroxide and a comprehensive computer analysis of their porous structure and adsorption properties based on benzene (C_6_H_6_) adsorption isotherms were carried out. In particular, the influence of the mass ratio of the activator’s dry mass to the char mass on the formation of the microporous structure of the obtained activated carbons was analysed. The summary of the analyses carried out based on benzene adsorption isotherms begged the conclusion that activated carbon with a maximum adsorption volume in the first adsorbed layer and homogeneous surface can be obtained from ebony wood at a mass ratio of the activator to the char of *R* = 3. The obtained results confirmed the superiority of the new numerical-clustering-based adsorption analysis (LBET) method over simple methods of porous structure analysis, such as the Brunauer–Emmett–Teller (BET) and Dubinin–Raduskevich (DR) methods. The LBET method is particularly useful in the evaluation of the influence of the methods and conditions of production of activated carbons on the formation of their porous structure. This method, together with an appropriate economic analysis, can help in the precise selection of methods and conditions for the process of obtaining activated carbons at specific manufacturing costs, and thus makes it possible to obtain materials that can successfully compete with those of other technologies used in industrial practice and everyday life.

## 1. Introduction

Activated carbons are amorphous carbonaceous materials characterized by a large specific surface area and adsorption capacity. The production of activated carbons is usually carried out by physical activation after carbonisation, one-step physical activation, or chemical activation [1,2]. Carbonisation consists of the heating of a raw material either without access to air or in an inert gas atmosphere [3,4]. At an early stage of carbonisation, e.g., at about 300–350 °C, the less durable links in the polymeric network break up, and free macromolecules are formed. At the same time, substantial amounts of volatile components are discharged, and the atoms of carbon are regrouped into a more stable form [3,4]. Cross-links between the macromolecules are also generated, and the stiff, network-like carbonaceous structure that develops in the process reflects the development of the microporosity of the material. When the carbonisation temperature exceeds 500 °C, the structure becomes increasingly carbonised and aromatised as a result of the elimination of the atoms of hydrogen and oxygen [3,4]. 

The properties of the product of carbonisation are determined by such factors as the end temperature of the carbonisation, the processing time, the rate of temperature increase, and the processing atmosphere [3,4]. The end temperature of carbonisation is one of the most important parameters of the process because a substantial amount of energy must be delivered in order to break up the less durable links, distil the volatile products of the thermal decomposition of the raw material, and organise a dense carbonaceous substance [3,4]. The parameters of the primary porous structure developed by way of carbonisation are insufficient for the majority of adsorption processes. In order to achieve further development of the structure of the pores, the char undergoes additional processing, i.e., activation.

Activation processes are generally divided into two types—namely, physical and chemical activation [5]. Physical activation consists of partial gasification of the char with oxidising agents, e.g., water vapour or carbon dioxide at a temperature of 800–1000 °C, or, less frequently, oxygen at a temperature below 800 °C; the use of a mixture of these agents is also possible [6,7,8]. The reactions taking place in the process bring about the production of gaseous products and a gradual reaction of the carbonaceous substance, which is replaced with empty spaces known as pores, while the specific surface area of the material increases. Temperature has the highest impact on the course of the activation process; at relatively low temperatures, the rate of the chemical reaction of the char with the oxidising agent is low, as is the rate of the whole process [6,7,8].

The production of activated carbons by way of chemical activation consists of a one-step reaction of the raw material with the activating agents [9,10,11,12]. Compared to physical activation, chemical activation helps lower the temperature of the process and increase its efficiency. The chemical activation process usually yields carbon adsorbents that feature a more developed porous structure with a narrower pore width distribution and a considerably larger specific surface area compared to the results of physical activation [9,10,11,12]. However, the chemical activation process is more expensive than physical activation, and it requires an additional rinsing step. In addition, the chemical compounds used in chemical activation are highly corrosive and, in particular, zinc chloride has a negative impact on the natural environment; hence, this activator is no longer used in industry.

The porous structure and functional properties of carbonaceous adsorbents are also dependent on the structure of the original raw material. As a consequence, the choice of a suitable material is no less important than the selection of an adequate production technology and the determination of the optimum processing conditions. Therefore, a search for new raw materials that would be useful in the production of carbonaceous adsorbents has been under way, and particular attention has been paid in this regard to biomass waste from the food, timber, and wood industries and agriculture [13,14,15,16,17,18,19,20,21].

The wood processing industry, the carpentry industry, and other related industries have a huge potential in terms of raw materials; hence, the production of activated carbons from biomass waste could increase the economic return and reduce pollution. This is connected with the fact that the production of adsorbents is not only one of the alternative methods for utilising biomass waste, but is also a way of converting such waste into a valuable and sustainable product [13,14,15,16,17,18,19,20,21].

## 2. Materials and Methods

Considering the increasing demand for carbonaceous adsorbents derived from biomass, a study was carried out that included the preparation of activated carbons from hard biomass types by potassium hydroxide activation and a comprehensive analysis of their porous structure and adsorption properties. The activated carbons were derived from mahogany wood (MA), ebony wood (EB), hornbeam wood (HB), pistachio nut shells (PO), hazelnut shells (HT), and pecan nut shells (PN), from which 1–3.15 mm grain fractions were prepared and subjected to the process of carbonisation at 500 °C. The char obtained from each of the raw materials was mixed with solid potassium hydroxide powder (Sigma-Aldrich Chemie GmbH, Munich, Germany) and crushed in a mortar to homogenise the mixtures. For the purpose of the study, samples of activated carbons were obtained according to the ratio of the dry mass of the activator to the mass of the char, i.e., the mass ratios of *R* = 1, 2, and 3. In preliminary studies, higher amounts of KOH were also considered; however, rapid degradation of the porous structures of activated carbons obtained from wood occurred for mass ratios above *R* = 3. After impregnation with potassium hydroxide, the samples were heated in a vertical tube furnace (Wrocław University of Technology, Wrocław, Poland) under a nitrogen flow (30 dm^3^/h) at a rate of 5 °C/min until the final activation temperature of *T* = 800 °C. The activated carbon samples were subsequently kept at the final activation temperature for 1 h, then cooled to room temperature under the nitrogen flow. The activated carbons obtained were then treated with 0.5 M HCl (Sigma-Aldrich Chemie GmbH, Munich, Germany) solution and washed with hot distilled water to wash out the chloride ions. The final stage of preparation of the samples consisted of drying the activated carbons at a temperature of 100 °C to a constant weight.

Despite the widespread use of nitrogen in the analysis of adsorption processes, it is increasingly stressed that the use of nitrogen in adsorption studies, especially in microporous materials, due to the hysteresis effects observed when using nitrogen adsorption, leads to erroneous estimates of surface area, among other things. Therefore, the concept of conducting the study using benzene, which has been proven for many years in adsorption studies, was developed. This adsorbate enables more reliable analysis of carbon microporous materials, compared to the commonly used nitrogen. For the activated carbons obtained in the present study, benzene adsorption isotherms were determined at a temperature of 25 °C using the gravimetric method (Wrocław University of Technology, Wrocław, Poland).

Based on the above-mentioned benzene adsorption isotherms, the following parameters were determined: the specific surface area *S_BET_*, which was calculated via the Brunauer–Emmett–Teller (BET) equation [22], the volume of micropores *V_DR_*, as calculated via the Dubinin–Radushkevich (DR) equation [23], the total volume of pores *V_T_,* as calculated via the maximum benzene adsorption at *P*/*P*_0_ = 0.96, as well as the volume of the first adsorbed layer *V_hA_* (cm^3^/g), the energy parameter for the first adsorbed layer *Q_A_*/*RT,* the energy parameter for the higher adsorbed layers *B_C_*, the geometrical parameters of the porous structure that determined the height and width of the adsorbate molecule clusters (*α* and *β*, respectively), the surface heterogeneity parameter *h*, the adsorption energy distributions on the first layer, and the identification index *w_id_,* which was obtained via a new numerical-clustering-based adsorption analysis (LBET) method [24,25,26,27].

As part of the research in this study, the SEM-EDX method was used to analyse the elemental compositions of the raw materials, i.e., mahogany wood (MA), ebony wood (EB), hornbeam wood (HB), pistachio nut shells (PO), hazelnut shells (HT), and pecan nut shells (PN), as well as the activated carbons obtained from these materials via chemical activation with potassium hydroxide at the impregnation ratio of *R* = 3. An SEM-EDX analysis was performed by using an energy-dispersive X-ray spectrometer Quantax 200 (Bruker AXS, Madison, USA) with an EDX detector XFlash 4010 (Bruker AXS, Madison, WI, USA). The mentioned detector was coupled with a scanning electron microscope LEO 1430VP (Carl Zeiss AG, Oberkochen, Germany). For the EDX detector, which was used to study the quantitative composition of the elements, in the analysis, the accuracy was 0.5, the sensitivity was 500–100 ppm, and the maximum depth, i.e., the impact area from which the registered X-ray was obtained, was in the range of 2 to 5 µm. As part of the research, imaging was also performed with the use of a scanning electron microscope Quanta 3D FEG (Fei, Waltham, MA, USA) with field emission. Observation of the analysed samples was performed in a high vacuum at an accelerating voltage of 20 or 10 kV with a secondary electron detector. 

## 3. Discussion of the Obtained Results

### 3.1. The Results of the Analysis of the Benzene Adsorption Isotherms

**Figure 1:**
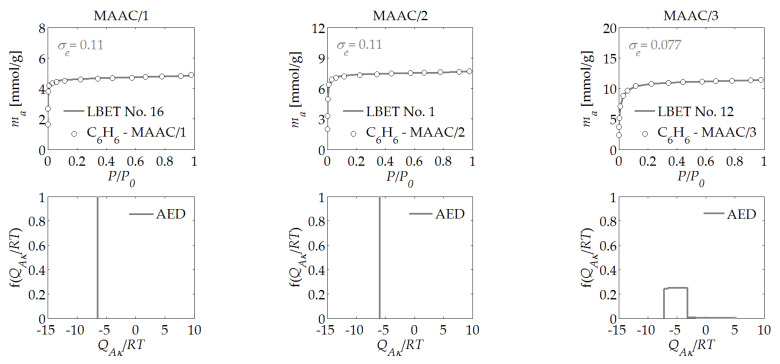
The benzene adsorption isotherms and the results of the identification of the adsorption systems via the LBET method, and adsorption energy distributions (AEDs) obtained for the MAAC/*R* active carbons, which were derived from mahogany wood.

**Figure 2:**
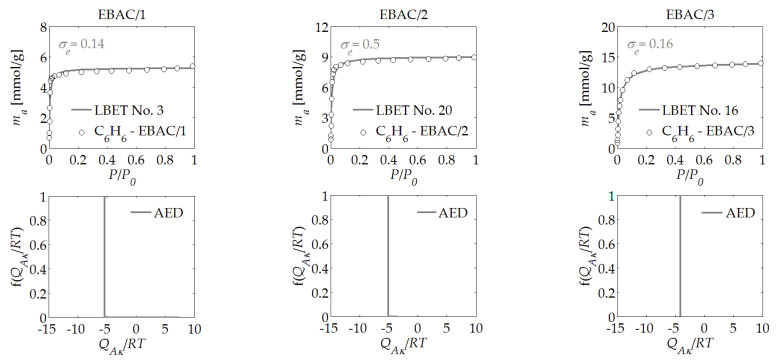
The same as Figure 1, but for the EBAC/*R* activated carbon derived from ebony wood.

**Figure 3:**
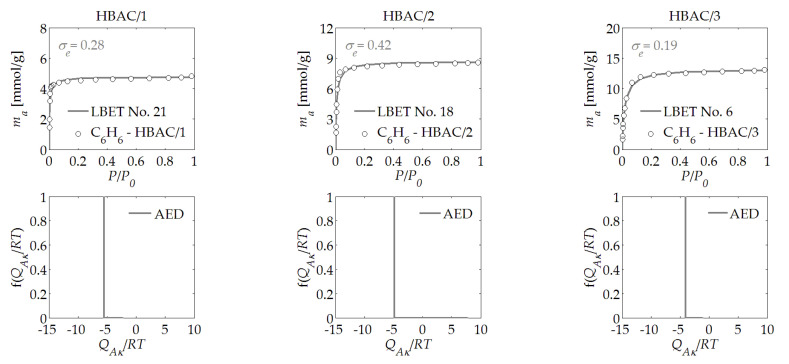
The same as Figure 1, but for the HBAC/*R* activated carbons derived from hornbeam wood.

**Figure 4:**
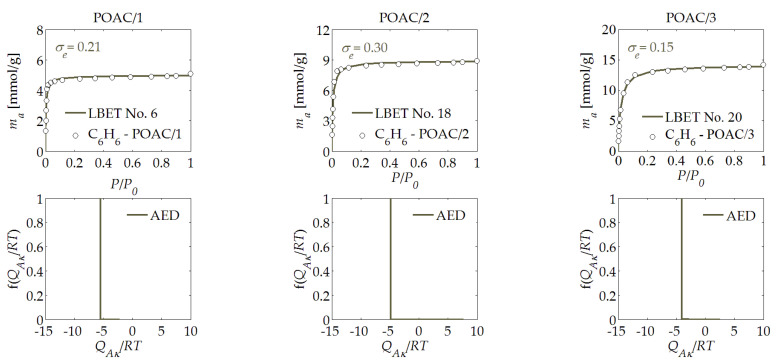
The same as Figure 1, but for the POAC/*R* activated carbon derived from pistachio nut shells.

**Figure 5:**
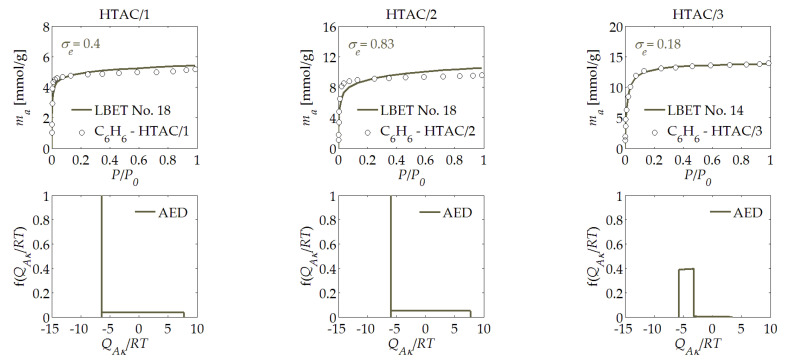
The same as Figure 1, but for the HTAC/*R* activated carbon derived from hazelnut shells.

**Figure 6:**
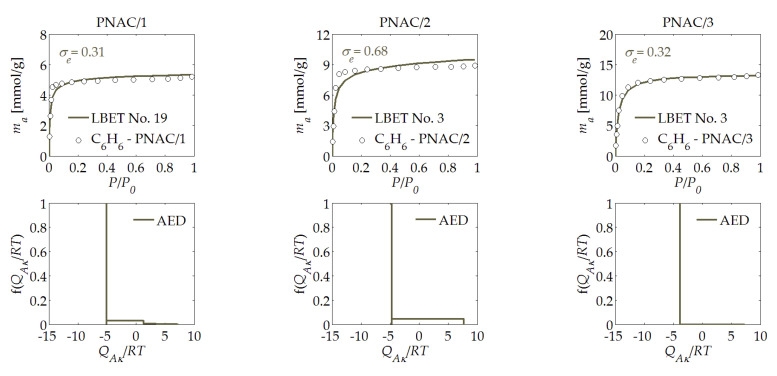
The same as Figure 1, but for the PNAC/*R* activated carbon derived from pecan nut shells.

The results of the analysis of the benzene adsorption isotherms are presented in Table 1, and in Figure 1, Figure 2, Figure 3, Figure 4, Figure 5 and Figure 6. The obtained results show a very high potential for the production of activated carbons from waste of biomass through the activation process using potassium hydroxide, as they featured a very large specific surface area and micropores volume.

Summarized in Table 1, the results of the analyses carried out on the benzene adsorption isotherms obtained at 298 K allow a conclusion that, with the increase in the mass of the activator compared to the mass of the char, the values of the parameters *S_BET_*, *V_DR_*, and *V_T_* for all analysed activated carbons gradually increased. Consequently, the highest values of *S_BET_*, *V_DR_*, and *V_micro_* were obtained for all activated carbons derived at the activator to char mass ratio of *R* = 3. In turn, among all of the activated carbons derived, the highest values for the structural parameters—i.e., *S_BET_*, *V_DR_*, and *V_T_*—was obtained for the HTAC/3 activated carbons, which were derived from hazelnut shells.

It should be stressed, however, that these values did not significantly differ in comparison to the other activated carbons derived, with the exception of the MAAC/*R* sample. However, the information obtained with the BET [22] and DR [23] methods did not give a complete picture of the analysis of the porous structure, including the information about the surface heterogeneity, and such information is essential for the production of high-quality activated carbons that can be dedicated to specific advanced adsorption processes. More information on the porous structures of the derived activated carbons was obtained by using the LBET method [24,25,26,27].

The results obtained based on the adsorption isotherms of benzene on the MAAC/1 activated carbon that was derived from mahogany wood at the activator to char mass ratio of *R* = 1 indicated that the surface of the material was homogeneous (*h* = 0) and that very high and branched clusters of molecules of benzene were formed in its micropores. The development of clusters of molecules of the adsorbate was blocked by the limited geometry of the micropores, as indicated by the type of the best-fitted LBET model. For the MAAC/1 sample, a relatively small value was obtained for the volume of the first adsorbed layer, i.e., *V_hA_* = 0.409 cm^3^/g. However, the very high value of the energy parameter for the upper layers, *B_C_* = 35.00, was notable, as it indicated a strong impact of adsorption in the layers above the first, which confirmed the earlier observations. The shape of the energy distribution of the adsorption on the first layer, which was determined on the basis of the benzene adsorption isotherms (see Figure 1), indicated the availability for benzene molecules in the adsorption sites of the analysed material with a practically identical adsorption energy. From the shape of the energy distribution on the first layer and the values of the geometrical parameters, it can be concluded that high-branching clusters of adsorbate molecules were formed in the pores of this material.

The analysis of the results obtained for the MAAC/2 sample, which was derived at the mass ratio of *R* = 2, demonstrated that there was only a slight increase in the volume of the first adsorbed layer, i.e., *V_hA_* = 0.568 cm^3^/g. In addition, the significantly different values of the geometric parameters were noticeable, which indicated that, in the pores of the studied material, clusters of one molecule in height were formed, but they were branched. On the other hand, the shape of the energy distribution on the first layer that was determined for the MAAC/2 sample indicated the occurrence of a very narrow range of energy on its surface and, thus, practically the same energy conditions at the potential adsorption sites. 

In the case of the analysis of the MAAC/3 sample, which was derived from mahogany wood, at the mass ratio of *R* = 3, there was a very large increase in the volume parameter in the first adsorbed layer in comparison with the sample obtained at *R* = 2, but with a rapid increase in the heterogeneity of the surface from *h* = 0—i.e., a homogeneous surface—to *h* = 7—i.e., strongly heterogeneous surface. It was also noteworthy that the limitations in the growth of the clusters of molecules of the adsorbate were, in this case, of a geometric nature. The shape of the energy distribution on the first layer that was determined for the MAAC/3 sample (see Figure 1) indicated the occurrence of a relatively wide range of energy on its surface, which allowed us to conclude that, in this case, there was a significant non-energetic uniformity of the surface. On the basis of this information, it can be concluded that, in the case of the MAAC/3 sample, uncontrolled burning of the material’s surface occurred, which was caused by the use of too much activator.

In the case of the analysis of the benzene adsorption isotherms that were determined for the EBAC/*R* activated carbons derived from ebony wood, it was demonstrated that, with the increase in the mass ratio of hydroxide to char, the value of the first adsorbed layer (*V_hA_*) gradually increased—as did the value of the energy parameter for the upper layers (*B_C_*)—with the decreasing value of the adsorption energy for the first layer (*Q_A_*/*RT*). Moreover, the results of the analysis of the sample EBAC/3, which was derived from ebony wood at the mass ratio of *R* = 3, demonstrated that the surface of the material was homogeneous, despite the fact that the samples derived at a lower mass ratio of hydroxide to char demonstrated the heterogeneity of the surface. Moreover, in contrast to the samples obtained at lower mass ratios, high clusters of molecules of benzene were formed in the micropores of the sample, as indicated by the value of the parameter *α*, which indicated that, in the case of the activated carbon sample designated as EBAC/3, the porous structure was fired deep into the material, resulting in a significant increase in the height of the micropores.

The type of the best-fitted model pointed to a limit in the growth of the clusters of molecules of the adsorbent due to the limited pore geometry. On the other hand, the shapes of the energy distributions on the first layer determined for EBAC/*R* activated carbon (see Figure 2) indicated the occurrence of adsorption sites of practically equal adsorption energies in the material under study, irrespective of the amount of activator used, i.e., potassium hydroxide. On this basis, a conclusion could be drawn about the significant influence of the original structure of the raw material on the formation of the porous structures of the activated carbons obtained from it. On the basis of preliminary studies, an increase in the ratio of the activator to the char to the value of *R* = 4 was considered; however, in the case of the activated carbons derived from wood, the burning of the external surfaces of the char grains was observed. Therefore, having in mind one of the objectives of this work, i.e., the comparison of adsorption properties of activated carbons derived from different types of biomass, the mass ratio above 3 was not considered here.

The next materials analysed were the samples of activated carbons derived from hornbeam wood at different activator to char mass ratios, i.e., the mass ratio *R*. In the case of the HBAC/*R* activated carbons, the volume of the first adsorbed layer and the energy value parameter for the upper layers increased with the increase in the mass ratio. At the same time, the value of the energy adsorption on the first layer and the values of the geometrical parameters *α* and *β* decreased. In the case of the HBAC/*R* activated carbons, virtually identical degrees of surface heterogeneity and shapes of the adsorption energy distribution were obtained for all of the samples at different ratios *R* (see Figure 3).

The next activated carbons analysed were the POAC/*R* samples, which were obtained from pistachio nut shells. Based on the results of the benzene adsorption isotherm analysis, which are shown in Table 1, it can be concluded that the POAC/1 activated carbons that were derived from pistachio nut shells at the mass ratio of *R* = 1 featured a moderate volume of the first adsorbed layer *V_hA_*. On the other hand, the values of the energy parameters for the first layer and other layers suggest the existence of preferential conditions for the adsorption of individual adsorbate molecules, which was also indicated by the values of the geometric parameters *α* and *β*. The number of the best-fitted LBET model, i.e., No. 22, indicated that the dominant limitations for the development of clusters of benzene molecules resulted from competitive adsorption. 

The shapes of the energy distributions on the first layer determined for the POAC/*R* activated carbons (see Figure 4) indicated the occurrence of adsorption sites with practically the same adsorption energies, irrespective of the amount of activator used, similarly to the case of the EBAC/*R* samples. In turn, the POAC/2 activated carbons prepared at the mass ratio of hydroxide to char of *R* = 2 featured a much larger volume of the first adsorbed layer, as well as a lower degree of surface heterogeneity; at the same time, the other parameters were very similar to the parameters of the sample obtained at *R* = 1. For the POAC/2 activated carbons, the number of the fitted LBET class model indicated that the limitations of the development of clusters resulted from the geometric limitations of the pores and the high adsorption energy in layers higher than the first layer. The sample of the POAC/3 activated carbons produced at *R* = 3 featured a significantly higher value of the *V_hA_* parameter, while the values of the energy and geometric parameters also indicated a higher preference for the formation of clusters of benzene molecules by combining adjacent adsorbate molecules. The number of the LBET class model suggested the occurrence of only geometric limitations to the growth of clusters of benzene molecules.

The subsequent stage of the research involved the analysis of the activated carbon samples derived from hazelnut shells, which were designated as HTAC/*R*; as discussed earlier, they were prepared with three different mass ratios, i.e., *R* = 1, 2, and 3. At first glance, the data collected in Table 1 show the difference from the POAC/*R* sample that was analysed before, whereby the HTAC/2 activated carbon sample that was prepared at the activator to char mass ratio of *R* = 2 featured a much higher value of the *V_hA_* parameter, i.e., the volume of the first adsorbed layer, as well as a lower surface heterogeneity, i.e., *h* = 2, and a greater height of the clusters of benzene molecules, i.e., *α* = 0.69.

For the HTAC/3 sample obtained at the mass ratio of *R* = 3; however, a decrease in the volume of the first adsorbed layer (*V_hA_*) could be observed, as well as a reduction of the height of the benzene molecule clusters and a significant increase in surface heterogeneity, as suggested by the value of the heterogeneity parameter *h* (*h* = 9).

The shapes of the energy distributions on the first layer determined for the HTAC/*R* activated carbons (see Figure 5) indicated for the HTAC/1 and HTAC/2 samples that these materials mainly contained adsorption sites with practically equal adsorption energies, as well as a small share of adsorption sites with very broad adsorption energy distributions. On the other hand, the HTAC/3 activated carbon sample, i.e., the one derived at the highest activator to char mass ratio, showed the occurrence of a relatively broad adsorption energy distribution for the first adsorbed layer. The results of the above calculations suggest that the material featuring the optimal adsorption properties was obtained from hazelnut shells at a mass ratio of *R* = 2. The further increase in the mass ratio led, primarily, to the increased heterogeneity of the surface, which resulted from the rapid and uncontrolled burning of the material’s surface.

The next stage of the research involved the analysis of the activated carbons derived from pecan nut shells, which were designated as PNAC/*R*; as discussed earlier, they were prepared with three different mass ratios of the activator to the product of carbonization, i.e., *R* = 1, 2, and 3. The results collected in Table 1 suggest that, in the case of the mass ratio of *R* = 1, the potential offered by the pecan nut shells was not fully utilised; significant development of a porous structure was only observed starting with *R* = 2, which was demonstrated by the volume of the first adsorbed layer (*V_hA_* = 1.258 cm^3^/g). Interestingly, at the same time and at the same mass ratio, the degree of surface heterogeneity also dropped to *h* = 2, unlike in the PNAC/1 sample obtained at *R* = 1. With the further increase in the mass ratio to *R* = 3, the shapes of the benzene molecule clusters changed, which was indicated by the values of the geometric parameters *α* and *β*.

For most of the activated carbon samples analyzed, the assessments of the adsorption energy distribution on the first layer determined based on the benzene adsorption isotherms, as presented in Figure 1, Figure 2, Figure 3, Figure 4, Figure 5 and Figure 6, showed a predominant share of adsorption points with a very narrow energy spectrum. A predominant share of adsorption points with a wider spectrum of adsorption energy was only observed for the HTAC/3 activated carbon sample, which was obtained at a mass ratio of *R* = 3. The shapes of the energy distributions on the first layer determined for the PNAC/*R* activated carbons (see Figure 6) indicated that, in these materials, there were primarily adsorption sites with practically the same adsorption energies and a small share of adsorption sites with very wide adsorption energy distributions. In the case of the PNAC/3 activated carbons, the proportion of the adsorption sites with very wide adsorption energy distributions was the smallest.

### 3.2. The Results of the Analysis of Elemental Composition

As part of this research, the elemental compositions of the raw materials and the activated carbons obtained from them were analysed via the SEM-EDX method. It could be observed that both the raw materials and the obtained activated carbons were characterised by similar contents of carbon (C) and oxygen (O). This was due to the fact that the types of raw materials used for the preparation of the activated carbons in the present study had similar physicochemical properties. Based on the SEM-EDX research, which was conducted for both the primary raw materials and the activated carbons derived from them presented in Table 2, it is correct to conclude that the highest content of C and O was found in pecan nut shells and the PNAC/3 activated carbons, which were obtained from pecan nut shells. The results obtained through the elemental analysis indicated that the percentage of elemental carbon was not the dominant factor in the determination of the development of the porous structure of the activated carbons under study.

### 3.3. The Results of the Analysis via Scanning Electron Microscopy

**Figure 7:**
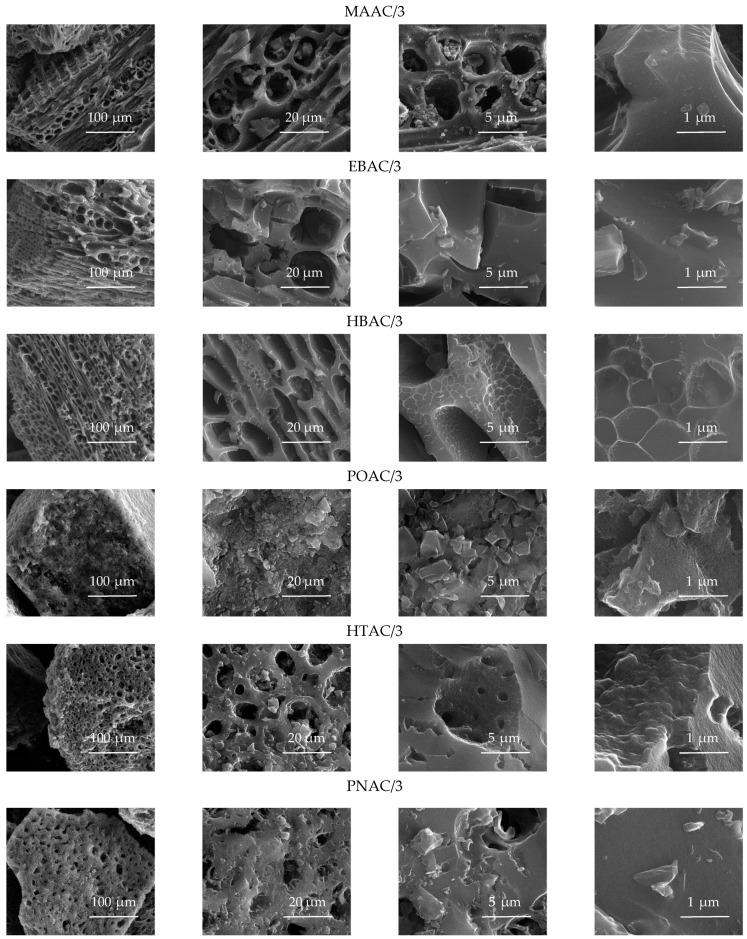
SEM images of the activated carbon samples obtained from various biomass materials.

The results of the investigations carried out using scanning electron microscopy showed a correlation between the morphology of the material from which the activated carbons were obtained and their adsorption properties. In particular, from the results of SEM imaging, significant similarities could be observed in the morphological structures of the activated carbons obtained from mahogany wood (MAAC/3), ebony wood (EBAC/3), and hornbeam wood (HBAC/3) (see Figure 7). 

The anisotropic cellular structure that is characteristic of wood was particularly evident. Based on the imaging performed using scanning electron microscopy, the structures of mahogany wood and ebony wood were found to be virtually identical, and the structure of hornbeam wood was very similar to those of the samples of African types of wood. This observation confirmed the results of the analysis of the porous structure of the activated carbons obtained from the aforementioned biomass materials.

The structures of the activated carbons derived from nut shells—e.g., pistachio nut shells (POAC/3), hazelnut shells (HTAC/3), and pecan nut shells (PNAC/3)—were definitely different. At first glance, in comparison with the activated carbons obtained from wood, one can see the isotropic structure and a significantly smaller proportion of voids between the walls, as well as the smaller diameters of the voids resulting from the much denser structures of the nut shells. These observations suggest the better adsorption properties of activated carbons derived from nut shells.

## 4. Conclusions

The results of the research presented in this article highlighted the significant potential for production of activated carbons with a very high adsorption capacity and large specific surface area from waste biomass by means of activation with potassium hydroxide. The activated carbons obtained as part of this research were, in fact, characterised by adsorption properties that were almost the same as those of the best carbonaceous adsorbents derived from homogeneous polymers. The results of the analysis of the porous structure of the activated carbons derived in this research showed that adsorbents with a maximum volume of adsorption on the first adsorbed layer and surface homogeneity can be obtained from ebony at the activator-to-char mass ratio of *R* = 3. Therefore, this begs the conclusion that the waste material of the timber, carpentry, and food industries can yield high-quality activated carbons and their derivatives, i.e., monoliths of activated carbons that can be widely applied in many technologies and in everyday life.

There are very complicated mechanisms that occur during the processes of the initial carbonisation and activation, and, thus, there is practically no possibility of predicting them. Therefore, in order to obtain adsorbents with appropriate structural parameters, it is necessary to perform a series of experiments supplemented by a reliable analysis of the structure by using advanced methods. It should be emphasised that the research projects presented here yielded a broad spectrum of information and shed new light on issues pertaining to the assessment of the effects of technologies for the production of carbonaceous adsorbents on the parameters of the microporous structures produced, and this was possible thanks to the application of the LBET method in the analyses. With the use of the LBET method, the analyses enabled the gathering of much more information on the microporous structures compared with what would be possible with the BET or DR method.

## Figures and Tables

**Table 1:** Parameters of the porous structure of activated carbons derived from mahogany wood (MAAC/*R*), ebony wood (EBAC/*R*), hornbeam wood (HBAC/*R*), pistachio nut shells (POAC/*R*), hazelnut shells (HTAC/*R*), and pecan nut shells (PNAC/*R*), as calculated from the isotherms of benzene.

*R*	*S_BET_* [m^2^/g]	*V_DR_* [cm^3^/g]	*V_T_* [cm^3^/g]	LBET No.	*V_hA_* [cm^3^/g]	*Q_A_*/RT	*B_C_*	*α*	*β*	*h*	*w_id_*
MAAC											
1	998	0.404	0.435	16	0.409	−6.46	35.00	1.00	1.82	0	0.98
2	1607	0.653	0.681	1	0.667	−5.94	6.74	0.04	3.40	0	0.99
3	2336	0.962	1.021	12	1.010	−7.16	7.07	0.03	3.55	7	0.97
EBAC											
1	1088	0.449	0.476	3	0.467	−5.40	27.22	0.009	1.01	2	0.91
2	1885	0.759	0.798	20	0.787	−5.03	34.57	0.007	1.05	3	0.89
3	2953	1.181	1.244	16	1.225	−4.12	35.00	0.70	1.00	0	0.93
HBAC											
1	992	0.403	0.428	21	0.422	−5.52	22.86	0.008	2.86	3	0.95
2	1819	0.728	0.766	18	0.773	−4.85	34.40	0.001	1.12	2	0.94
3	3005	1.103	1.166	6	1.176	−4.03	35.00	0.001	1.00	3	0.99
POAC											
1	1055	0.429	0.462	6	0.442	−5.49	34.07	0.00	1.05	3	0.57
2	2019	0.810	0.853	18	0.789	−4.88	32.92	0.00	1.07	2	0.81
3	3013	1.163	1.240	20	1.254	−4.06	5.06	0.00	1.65	3	0.62
HTAC											
1	1041	0.423	0.449	18	0.666	−6.42	35.00	0.52	1.00	2	0.63
2	1903	0.748	0.790	18	1.523	−5.95	35.00	0.69	1.00	2	0.55
3	3131	1.165	1.254	14	1.234	−5.71	1.30	0.01	1.07	9	0.36
PNAC											
1	1066	0.434	0.466	19	0.504	−5.06	35.00	0.21	1.01	3	0.61
2	1834	0.756	0.796	3	1.258	−4.67	35.00	0.53	1.00	2	0.58
3	2787	1.107	1.181	3	1.197	−3.82	22.64	0.00	1.16	2	0.47

**Table 2:** Elemental compositions of the raw materials and the activated carbons, as determined with SEM-EDX.

Sample	The Percentage of the Element, %			
	C	O	Al	Cu
M	20.63	63.62	0.50	0.28
MAC/3	41.67	56.46	1.52	0.35
E	21.73	69.61	3.43	0.31
EAC/3	40.20	54.38	3.67	0.42
HB	21.56	75.98	1.10	0.23
HBAC/3	41.49	55.31	2.48	0.34
PO	20.25	67.63	1.91	0.25
POAC/3	37.70	58.69	3.12	0.38
HT	22.39	75.68	1.10	0.22
HTAC/3	41.28	56.53	1.69	0.36
PN	24.28	68.64	1.42	0.24
PNAC/3	42.06	55.98	1.31	0.36

## Data Availability

The data presented in this work can be made available on request.
